# Irisin in the modulation of bone and cartilage homeostasis: a review on osteoarthritis relief potential

**DOI:** 10.3389/fphys.2025.1570157

**Published:** 2025-04-17

**Authors:** Mengtong Zhang, Wengieng Xiong, Ruohan Qiao, Minhan Li, Chuhan Zhang, Chi Yang, Yan Zhu, Jiaying He, Zhigui Ma

**Affiliations:** Department of Oral Surgery, Shanghai Ninth People’s Hospital, College of Stomatology, Shanghai Jiao Tong University School of Medicine, National Clinical Research Center for Oral Diseases, Shanghai Key Laboratory of Stomatology & Shanghai Research Institute of Stomatology, Shanghai, China

**Keywords:** irisin, FNDC5, osteoarthritis, bone metabolism, cartilage metabolism

## Abstract

Osteoarthritis, a progressive and degenerative joint disease, disrupts the integrity of the entire joint structure, underscoring the urgency of identifying more effective therapeutic strategies and innovative targets. Among these, exercise therapy is considered a key component in the early management of osteoarthritis, functioning by stimulating the secretion of myokines from the skeletal muscle system. Irisin, a myokine predominantly secreted by skeletal muscle during exercise and encoded by the FNDC5 gene, has garnered attention for its regulatory effects on bone health. Emerging evidence suggests that irisin may play a protective role in osteoarthritis by promoting tissue homeostasis, enhancing subchondral bone density and microstructure, and inhibiting chondrocyte apoptosis. By improving chondrocyte viability, preserving extracellular matrix integrity, and maintaining homeostasis in osteoblasts, osteoclasts, and osteocytes, irisin emerges as a promising therapeutic target for osteoarthritis. This review delves into the role of irisin in osteoarthritis pathogenesis, highlighting its influence on cartilage and bone metabolism as well as its dynamic relationship with exercise. Additionally, this review suggests that further exploration on its specific molecular mechanisms, optimization of drug delivery systems, and strategic utilization of exercise-induced benefits will be pivotal in unlocking the full potential of irisin as a novel intervention for osteoarthritis.

## 1 Introduction

Osteoarthritis (OA) is a chronic, progressive, and debilitating degenerative disease recognized as one of the most prevalent forms of arthritis. It is marked by complex pathological changes across the joint including articular cartilage damage, subchondral bone alterations, synovial tissue proliferation, enhanced vascularity, and instability of tendons and ligaments (Yao et al., 2023). Between 1990 and 2019, the prevalence of OA rose sharply, with an especially significant increase during the early 2000s. This trend is largely attributed to population growth and the aging trend, suggesting that OA incidence will continue to rise in the coming decades (Cao et al., 2024). Current global treatment guidelines for OA are primarily aimed at symptom relief and limitation of disease progression. Although no pharmacological treatment currently exists that completely cures OA, treatment strategies are gradually shifting towards early prevention, targeting the delay of disease progression before extensive joint destruction occurs (Yao et al., 2023).

Exercise therapy is considered a key component in the management of OA, with its capacity to alleviate pain and improve physical function by increasing skeletal muscle activity (Hunter and Bierma-Zeinstra, 2019). By stimulating the secretion of myokines in response to exercise, skeletal muscle can function as a secretory organ, which offers a novel insight into the benefits of exercise for OA treatment (Ning et al., 2022). In 2012, irisin was first identified as an exercise-induced myokine. It is derived from the fibronectin type III domain-containing protein-5 (FNDC5) gene product through protease cleavage. During exercise, peroxisome proliferator-activated receptor-gamma coactivator (PGC)-1α, released by skeletal muscle, contributes to the conversion of FNDC5 into irisin, which then enters the bloodstream and exerts its effects throughout the body (Boström et al., 2012). Exercise-induced irisin may play a significant role in alleviating the progression of OA. Moderate-intensity treadmill exercise increased irisin levels, exhibiting a relatively complete and smoother cartilage surface in a rat model of OA, as evidenced by the counteracting effect of irisin-neutralizing antibodies (Jia et al., 2022). The secreted fragment of irisin is highly conserved across all sequenced mammals, with 100% sequence identity between mouse and human irisin, underscoring its potential in shared biological processes and therapeutic targets (Boström et al., 2012). Irisin levels in the serum and synovial fluid (SF) correlated negatively with radiographic severity and Kellgren and Lawrence (KL) knee OA classification (Mao et al., 2016). In the DMM (Destabilization of the Medial Meniscus) OA mouse model, local injection of irisin reduces cartilage erosion and synovitis, enhancing joint integrity and gait patterns in injured knees (Wang et al., 2020). These results underscore the promise of irisin in OA management and the need for a deeper comprehension of its mechanism of action.

**FIGURE 1:**
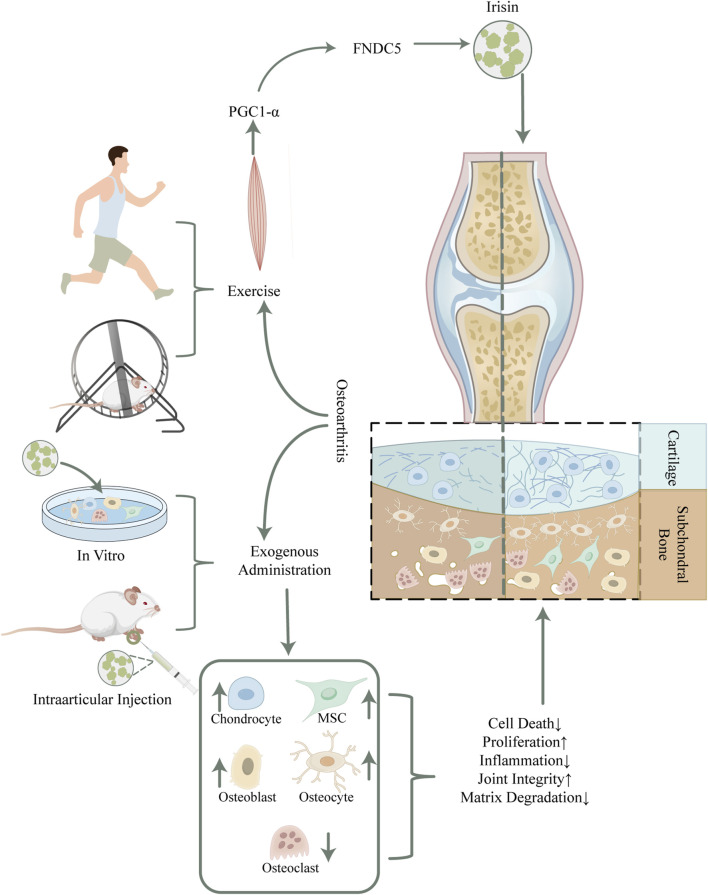
Schematic overview of potential mechanisms of irisin in mitigating osteoarthritis. Irisin, secreted by skeletal muscles during physical activity, has shown potential in reducing osteoarthritis symptoms in humans and rodents. The detailed mechanism has been unraveled by exogenous irisin intervention, which modulates the function and homeostasis within bone cells by binding to the αV/β5 integrin receptor, thus improving articular cartilage integrity and restoring subchondral bone mass. Abbreviations. PGC-1α, peroxisome proliferator-activated receptor gamma coactivator-1 alpha; FNDC5, fibronectin type III domain-containing protein-5; MSC, Mesenchymal Stem Cell.

This review reveals the correlation between irisin levels and OA severity, highlighting its potential in cartilage protection and bone tissue repair (Figure 1). A thorough examination of the impact of irisin on OA in the context of cartilage and subchondral bone metabolism was presented, with the hope of providing insights that will clarify the therapeutic effects of irisin and inform future research and applications.

## 2 Exercise and irisin

Irisin is an exercise-induced myokine, and many studies have demonstrated that exercise significantly increases irisin levels (Liu L. et al., 2021) (Table 1). For instance, circulating irisin levels in sedentary adult males were approximately 3.6 ng/mL as measured by mass spectrometry; however, these levels increased to approximately 4.3 ng/mL in aerobically trained individuals (Jedrychowski et al., 2015). Li et al. conducted studies based on *in vitro* and *in vivo* models, revealing that various forms of physical activity, including aerobic, resistance, and vibration exercise, as well as electrical stimulation of skeletal muscle, upregulated the expression of irisin/FNDC5 in the myocardium of mice (Li H. et al., 2021). This upregulation promotes mitophagy and enhances antioxidant function, ultimately leading to an improvement in cardiac function, particularly with a more pronounced effect observed during resistance exercise (Li H. et al., 2021).

**TABLE 1:** *In vivo* effects of irisin in alleviating osteoarthritis.

Model	Subjects	Dosage	Effects	References
Clinical model	Patients with knee OA (n = 215)	Not applicable	Irisin levels in serum and synovial fluid inversely correlate with radiographic KL severity	Mao et al. (2016)
	Aerobic interval training group (n = 6 males, 25 ± 5 years); Separate sedentary group (n = 4 males,26 ± 3 years)	12-week of high-intensity aerobic training	Human irisin circulates at ∼3.6 ng/mL in sedentary individuals; this level is increased to ∼4.3 ng/mL in individuals undergoing aerobic interval training	Jedrychowski et al. (2015)
Animal model	Twelve-week-old adult male C57BL/6 J wide type mice (n = 12) with OA surgically induced by ACLT	Not applicable	Rescued OARSI scoring	Li et al. (2021b)
			Improved thickness of cartilage	
	C57BL6 mice	100 μg/kg body weight; 4 weeks	Alleviated cartilage degradation; Decreased MMP-13, Caspase-3, BAX; Increased Trabecular bone number and BV/TV	He et al. (2020)

Abbreviations: OA, osteoarthritis; ACLT, anterior cruciate ligament-transverse; KL, kellgren and lawrence; MMP-13, matrix metallopeptidase 13; Caspase-3, cysteinyl aspartate specific proteinase-3; BAX, Bcl-2-associated X; BV/TV, bone volume/tissue volume; OARSI, osteoarthritis research society international.

Although most studies confirm that irisin is significantly upregulated during exercise, the mechanism of its upregulation and its transportation from myotubes to bone tissue is still not fully understood. Recently, Tao et al., (2024) demonstrated that exercise enhances the expression of irisin upstream protein PGC1-α by upregulating polyunsaturated fatty acids (arachidonic acid and docosahexaenoic acid), which, in turn, increases irisin secretion in skeletal muscle. In addition, this study revealed the mechanism involved in the transport of irisin from skeletal muscle to bone, i.e., muscle-derived irisin is transported *in vivo* using exosomes as carriers and enters osteoblasts in bone tissue via caveolin-mediated endocytosis which is dependent on Caveolin-1. But according to Pekkala et al. (2013), alterations in FNDC5 expression did not correlate with serum irisin, which indicates that there are other routes besides transcription that contribute to muscle irisin release. The entire process by which irisin is produced is still unclear and needs more research.

The impact of exercise on irisin is influenced by various factors, including the type and duration of exercise as well as the specific population being studied. Serum irisin levels were compared in detail across exercise phases in a 2014 study that involved subjects who were trained at varying levels in two distinct types of exercise (sprinting and cycling) (Huh et al., 2014). According to the researchers, irisin expression was irrespective of the kind of acute exercise and the individuals’ training state. However, running produced an extended period of heightened irisin compared to cycling. Pekkala et al. (2013) found that while a 21-week endurance training program and 1 h of low-intensity aerobic exercise did not significantly affect PGC1-α and FNDC5 mRNA levels in middle-aged men’s skeletal muscle, a single session of acute, high-intensity resistance exercise boosted FNDC5 mRNA expression by 1.4-fold. During 1 hour of acute high-intensity resistance exercise, the same study observed that PGC1-α mRNA alterations in skeletal muscle were significantly higher in young and older male subjects than in middle-aged males. According to the aforementioned research, exercise-induced elevations in irisin levels are beneficial for building bone health and muscle strength (Pekkala et al., 2013). However, when high-intensity exercise accompanied by excessive mechanical stress, not only poses a risk of joint damage but also has the potential to diminish the protective effects of irisin. For instance, Liu et al. (2016) established a rat model of exercise-induced OA, revealing that excessive pressure causes abnormal biochemical signals and Wnt/β-catenin pathway activation, leading to OA. In the context of articular cartilage injury, sustained high-intensity exercise increases catabolic protein activity over anabolic proteins, causing metabolic imbalance and OA induction. Furthermore, Rojas-Ortega et al. (2015) reported an increased expression of IL-1β in the articular cartilage when structural damage is still absent (3 days after the initiation of high-impact exercise). This finding helps to elucidate the onset of cartilage deterioration in advanced stages of OA. IL-1β directly inhibits the expression of cartilage-specific ECM genes (Goldring et al., 1988), upregulates matrix-degrading proteases (Richardson and Dodge, 2000), stimulates the production of reactive oxygen species (ROS), and induces chondrocyte death, thereby exacerbating cartilage degradation (Kourí et al., 1997).

The beneficial effects of irisin provide a scientific rationale for the development of targeted exercise therapies. However, to achieve optimal therapeutic outcomes, the selection of appropriate exercise modalities and intensities is of paramount importance (Kong et al., 2022). When devising exercise programs for patients with OA, it is imperative to consider individual differences and tailor the form, intensity, and frequency of exercise accordingly. Only through such personalized approaches can the therapeutic potential of exercise be maximized, ultimately yielding the greatest clinical benefits for patients.

## 3 FNDC5 gene and bone metabolism

Gene knockout (KO) and overexpression research in animal models demonstrate that alterations in FNDC5 levels can substantially affect critical cellular components involved in bone metabolism and their associated signaling pathways, indicating the regulatory function of irisin at the genetic level.

### 3.1 Gene knockout

At the cellular level, FNDC5 KO leads to increased bone resorption and reduced bone formation. Irisin-deficient mice created by FNDC5 KO exhibited increased osteoclast numbers (Luo et al., 2020), with higher expression of matrix metalloproteinase-9 (MMP-9) in both bone and adipose tissue (Zhu et al., 2021). Gene expression associated with osteogenesis in osteoblasts were also reduced in KO mice, including Receptor Activator for Nuclear Factor-κB Ligand (RANKL) (Luo et al., 2020), Runt-related transcription factor 2 (Runx2), special AT-rich sequence-binding protein 2, bone sialoprotein (Bsp), collagen I (COL I), and alkaline phosphatase (ALP) (Zhu et al., 2021). Additionally, inflammation-related cytokines like IL-6 and TNF-α were elevated in these irisin-deficient mice (Luo et al., 2020). These underlying mechanisms help to explain ultimate effects of irisin on bone tissue microstructure and overall OA progression.

At the tissue level, bone mass reduction is consistently observed in FNDC5 KO mice (Zhu et al., 2021; Xue et al., 2022). Critical bone microstructural parameters, such as bone volume/tissue volume (BV/TV), trabecular bone thickness (Tb.Th), trabecular number (Tb.N), and bone mineral density (BMD), were all reduced by approximately one-third. Measurement with OASRI scoring system (Li X. et al. 2021) also suggested that FNDC5 KO mice could develop more severe OA. Delayed endochondral ossification in the epiphysis of the limbs and phalanges was observed in FNDC5 KO mice, indicating the necessity of optimal irisin levels for proper bone growth and development (Zhu et al., 2021).

### 3.2 Gene knock-in and overexpression

Numerous gene overexpression studies reveal the osteogenic potential of FNDC5. Liu C. et al. (2021) first reported the miR-135a-5p/FNDC5/Irisin/Integrin αV/SMAD1/5 axis in bone marrow mesenchymal stem cells (BMSCs) osteogenesis, verifying that overexpression of FNDC5 enhanced osteogenesis by promoting Runx2 protein synthesis. *In vitro* assays and H&E staining further confirmed increased bone regeneration in mice with elevated FNDC5 expression.

FNDC5 overexpression is also linked to the alleviation of OA symptoms. Chen et al. (2020) demonstrated in an animal model that FNDC5 overexpression significantly alleviated OA symptoms, evidenced by decreased cartilage degradation, lower fat secretion, reduced synovial hyperplasia, diminished swelling, and fewer bone spurs. Notably, these mice exhibited significant improvements in gait, which indicates enhanced joint function.

In addition to overexpression studies, knock-in (KI) approaches have provided further insights into the role of Irisin in cartilage biology. Li X. et al. (2021) discovered that in primary mouse chondrocytes isolated from FNDC5 KI mice, chondrocytes exhibited reduced expression of inflammatory factors (IL-1, IL-6, TNF-α) and mediators (COX2, iNOS) alongside upregulated cartilage anabolic markers (COL2a1, ACAN, SOX9) under inflammatory conditions These findings highlight the dual role of Irisin in promoting cartilage proliferation and matrix gene expression while inhibiting inflammation.

## 4 Irisin mitigates osteoarthritis through the modulation of bone metabolism

A characteristic feature of OA is the degeneration of the subchondral bone, which disrupts the mechanical stress distribution and exacerbates articular cartilage damage. The subchondral bone, located beneath the cartilage, provides both mechanical and nutritional support and is anatomically divided into two components: the subchondral bone plate (SBP) and the trabeculae. The SBP is a dense, porous calcified plate, while the trabeculae are cancellous bone structures that undergo continuous bone remodeling (Hu et al., 2021). Bone remodeling is an ongoing process encompassing both osteoblast-mediated bone formation and osteoclast-mediated bone resorption. At the remodeling site, osteoblasts recruit bone marrow and blood mononuclear cells to differentiate into osteoclasts, which resorb bone. Subsequently, osteocytes attract bone marrow mesenchymal stem cells and progenitor cells to differentiate further, eventually becoming osteoblasts that secrete osteoid to fill the cavity. The process concludes with mineralization of the osteoid, thus completing bone remodeling (Feng and McDonald, 2011). In the early stages of OA, an escalation in subchondral bone remodeling is accompanied by a reduction in bone density and mineralization, changes that often occur in tandem with or precede the initial degradation of articular cartilage (Yao et al., 2023). Irisin effectively boosts subchondral bone mass by promoting the osteogenic differentiation of BMSCs, increasing osteoblast count and activity, while curbing osteoclastogenesis and activity. Furthermore, irisin improves bone microstructure and quality through osteocyte activation, which provides better support for the articular cartilage and helps to decelerate OA progression (Li et al., 2021) (Table 2).

**TABLE 2:** Specific mechanisms that irisin ameliorates bone metabolism imbalance.

Cell type	Dosage	Effects	References
Mesenchymal stem cells (MSC)			
BMSCs	40 μM; 48 h	Elevated autophagy through upregulation of the Atg12-Atg5-Atg16 L complex	Chen et al. (2020)
		Activation of Wnt/β-catenin signaling pathway and osteogenic	
Human mesenchymal stem cells (hMSCs)	100 ng/mL; 7.14 days	Activation of the Rap1/PI3K/Akt signaling axis by up-regulating miR-125b-5p targeting SIPA1L2	Chen et al. (2022)
		Induction of chondrogenic differentiation	
Osteoblast			
MC3T3-E1 preosteoblast cells	0.1 μg/mL; 48 h	Activated the AMPK pathway promoted osteoblast proliferation	Tao et al. (2024)
		Ferroptosis inhibition	
Primary rat osteoblasts and MC3T3-E1 cells	100 ng/mL; 24 h	Promoted osteoblast proliferation and differentiation via the activation of the p38/ERKMAPK pathway	Qiao et al. (2016)
Osteoclast			
Pre-osteoclast RAW264.7 cells and mouse bone marrow monocytes	20 nM; 60 min, 4 days	Decreased expression of p-p65, IkBα and p-JNK protein Increased expression of p-p38 protein	Ma et al. (2018b)
Osteocyte			
MLO-Y4 cells	100 ng/mL; 8 and 24 h	Downgraded H_2_O_2_–induced apoptosis via the activation of the ERK pathway Increased Pdpn mRNA	Storlino et al. (2020)
		Decreased Sclerostin and Dkk1 mRNA	

Abbreviations: MSC, mesenchymal stem cells; Atg, autophagy-related gene; Rap1, ras-related protein 1; PI3K, phosphatidylinositol 3-kinase; Akt, protein kinase B; SIPA1L2, signal-induced proliferation-associated 1 like 2; AMPK, AMP-activated protein kinase; p-p65, phosphorylated p65; IkBα, inhibitor of nuclear factor kappa-B alpha; p-JNK, phosphorylated c-Jun N-terminal kinase; Pdpn, podoplanin; Dkk1, dickkopf-1.

### 4.1 Irisin and subchondral bone mass

Irisin stabilizes subchondral bone by enhancing BMD and mechanical strength, improving its microstructure and overall quality. This effectively reduces the risk of cartilage degeneration and ameliorates osteoarthritic conditions (Ning et al., 2022). Irisin serum levels have been found to correlate inversely with age and directly with femoral and vertebral bone mineral density in the elderly, indicating a potential role for irisin in maintaining bone health in older adults (Colaianni et al., 2021). A similar positive correlation between FNDC5 mRNA levels and BMD has also been observed in animal models (Kawao et al., 2018). The administration of irisin resulted in an increase in BMD in the proximal tibia and the fourth lumbar vertebra, a reduction in resorptive surfaces, enhanced bone formation, and a decrease in inflammatory cytokines TNF-α and IL-17 in the caudal and lumbar regions of hindlimb unloading mice (Metzger et al., 2020). These findings substantiate the application of irisin in addressing skeletal disorders marked by diminished subchondral bone mass and underscore the importance of elucidating the mechanisms of action essential for developing effective treatments for bone health.

Several studies using FNDC5 KO and KI models with abnormal irisin levels also demonstrate a positive association between irisin and bone mass. Zhu et al. constructed a model with targeted FNDC5/irisin KO in osteoblasts, revealing a significant reduction in BMD during development and adulthood, accompanied by delays in bone development and mineralization, which in turn lessened the attenuated effect of the increased bone thickness that running wheel exercise promoted in mice (Zhu et al., 2021). Shimonty et al. observed that irisin plays distinct roles in skeletal development and maintenance in mice, varying by sex. Female mice with FNDC5 KO displayed reduced tartrate-resistant acid phosphatase (TRAP) positive osteoblasts and smaller osteoclast lumen under conditions of lactation or low-calcium diet, suggesting partial bone preservation. In contrast, male mice with FNDC5 KO under low-calcium diet conditions exhibited more significant reductions in these parameters and more intense osteolysis, indicating increased bone loss (Shimonty et al., 2024). While irisin correlates positively with bone density, sustained high irisin expression may affect osteoblast activity, leading to bone loss. An FNDC5-transgenic mouse model was employed to illustrate that elevated irisin was tied to lower bone mass, BV/TV, cortical thickness (Ct. Th), and osteoblast count at 2 months, accompanied by a reduction in bone formation rate (BFR). However, these effects were not observed in 13-month-old mice (Estell et al., 2020). These results indicate that normal irisin levels play an essential part in bone development and mineralization, while abnormal irisin levels may reduce bone strength and quality, thereby accelerating OA progression.

### 4.2 Irisin and mesenchymal stem cells

**FIGURE 2:**
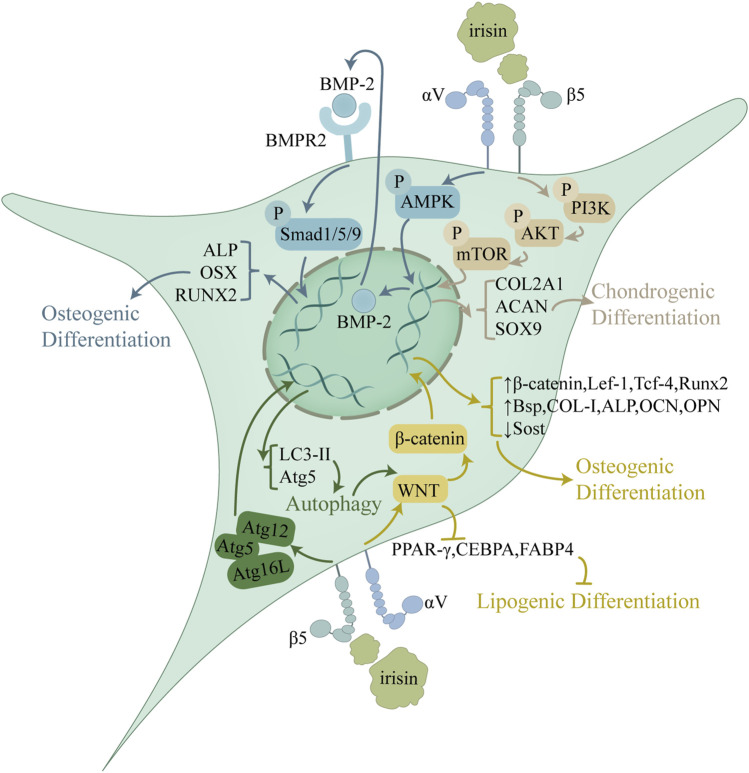
Role of irisin in the regulation of MSC proliferation and differentiation. Irisin upregulates autophagy through activation of the Atg12-Atg5-Atg16 L complex and activates the Wnt/β-catenin pathway, thereby inhibiting lipogenic differentiation and promoting osteogenic differentiation. Irisin exerts its regulatory effects on osteogenic differentiation also through the AMPK/BMP/SMAD pathway. In addition, Irisin promotes chondrogenic differentiation through the PI3K/Akt/mTOR pathway. By regulating the differentiation process of MSCs, Irisin mitigates metabolic disruptions in OA cartilage and subchondral bone, sustaining architecture and promoting repair.

The extensive cartilage damage overwhelms intrinsic reparative mechanisms in OA. Mesenchymal stem cells (MSCs), with their multilineage differentiation, are pivotal in the regeneration of cartilage and subchondral bone (Giorgino et al., 2023). Irisin augments MSCs chondrogenic and osteogenic differentiation, which is pivotal for cartilage and subchondral bone repair, thus attenuating OA advancement (Figure 2). This process consisted of the activation of the Atg12-Atg5-Atg16 L complex and an increase in levels of light chain 3 (LC3)-I/II and autophagy protein 5 (Atg5), both of which serve as markers of autophagy (Chen et al., 2020). Autophagy was downregulated in aged BMSCs and that autophagy played a vital role in maintaining the properties of BMSCs (Ma et al., 2018a). With increased autophagy, osteogenic differentiation and Wnt/β-catenin pathway activity enhanced, which led to upregulation of osteogenic genes like RUNX2, osteocalcin (OCN), and ALP, as well as key factors in the Wnt/β-catenin pathway, including β-catenin, lymphoid enhancer factor 1 (Lef-1), and T cell factor 4 (Tcf-4), while the expression of the osteogenic repressor sclerostin (SOST) was downregulated. Furthermore, the application of autophagy inhibitors validated that irisin depended on autophagy to enhance osteogenesis and was closely related to the Wnt/β-catenin pathway (Chen et al., 2020). By activating the Wnt/β-catenin pathway, irisin enhanced osteoblast activity and upregulated the expression of key osteogenic genes: RUNX2, Bsp, COL-I, ALP and Osterix (Osx). Osteoblastogenesis and mineralization followed as well. In addition, irisin could act on osteogenic differentiation by activating the p38 and ERK1/2 mitogen-activated protein kinase (MAPK) pathways, notably in combination with mechanical stretching (Zhang et al., 2019). Concomitantly, it was observed that irisin counteracted the suppressive effects of palmitate on osteogenic differentiation in BMSCs via the AMP-activated protein kinase (AMPK)/bone morphogenetic protein 2 (BMP2)/SMAD signaling pathway (Zhang et al., 2024).

In addition to promoting osteogenic differentiation, irisin effectively enhances the chondrogenic differentiation of MSCs. Irisin treatment at 100 ng/mL for 14 days increased the expression of miR-125b-5p, which targeted SIPA1L2 and consequently activated the Rap1/PI3K/AKT signaling pathway during chondrogenic differentiation in human MSCs (hMSCs). Along with enhancing the cartilage tissue quantity, this approach boosted the cartilage matrix production and upregulated the expression of the cartilage-specific markers COL2A1, ACAN, and SOX9 (Chen et al., 2022). In contrast, treatment of hMSCs with 20 nM irisin for 7 days impaired chondrocyte and osteocyte differentiation, which was evidenced by decreased expression of key genes critical for these processes, including osteopontin (OPN), Runx2 and collagenase XI (Di et al., 2024). Furthermore, the study indicated that irisin downregulated the expression of stem cell-specific genes, including OCT3/4, NANOG, and SOX2, which could interfere with stemness properties and drive their differentiation into specific cell lines (Di et al., 2024). Moreover, MSCs differentiation may follow a balanced process. By inhibiting adipogenic differentiation, irisin could steer more MSCs toward osteogenic and chondrogenic differentiation. Irisin modulates MSCs differentiation via Wnt/β-catenin pathways, promoting osteogenic differentiation while inhibiting adipogenic differentiation (Xing et al., 2025). This dual regulatory mechanism, further enhanced by mechanical stretch, may offer significant therapeutic potential for OA treatment by rebalancing MSCs fate determination toward bone and cartilage formation (Zhu et al., 2024).

Beyond directing MSC differentiation, irisin also stimulates the proliferation of these cells, thereby supplying a substantial pool of precursors for osteochondral tissue formation. Despite extensive research into the proliferative effects of irisin on MSCs, the literature has yielded inconsistent findings regarding its specific influence. The proliferative impact of irisin on BMSCs is likely contingent on both temporal and dosage factors, with possible detrimental effects emerging at high concentrations or with extended exposure (Chen et al., 2020). A comprehensive assessment of irisin as a therapeutic agent necessitates further studies to explore its effects and potential adverse outcomes on the proliferation and differentiation of various MSC subtypes (Di et al., 2024).

### 4.3 Irisin and osteoblasts

**FIGURE 3:**
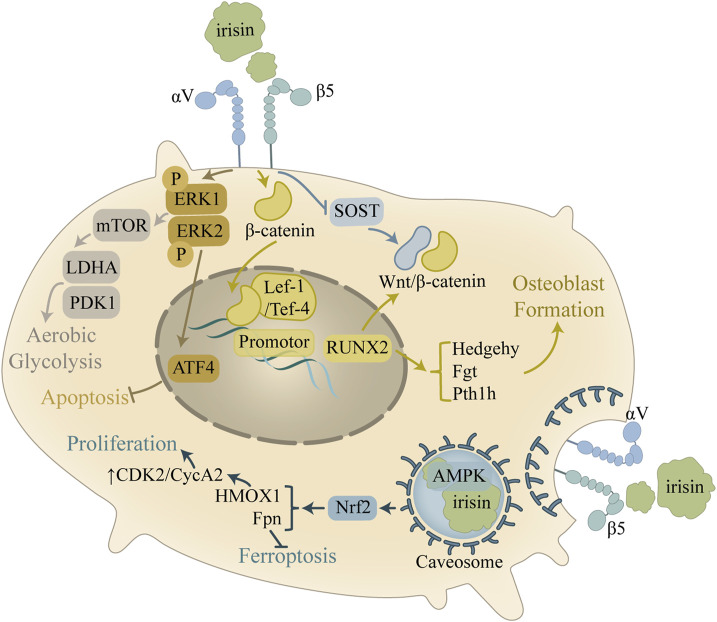
Mechanism of the improvement of osteoblast activity by irisin. In osteoblasts, the main role of irisin is to promote cell survival and osteogenesis. Irisin activates the AMPK pathway through caveosome formation, which regulates the cell cycle and inhibits iron death. Irisin also modulates the ERK signaling pathway and inhibits apoptosis by upregulating Atf4, in addition to regulating downstream pathways to promote glycolysis.

Irisin enhances osteoblast activity, bone formation, and osteogenesis through multiple signaling pathways, including Wnt/β-catenin, MAPK, and AMPK (Figure 3). The regulation effects of irisin on Wnt/β-catenin signaling pathway has been discussed in Section 4.2. Irisin and Mesenchymal Stem Cells (Chen et al., 2020). According to research by Qiao et al. (2016), irisin promotes the proliferation, differentiation, and *in vitro* mineralization of osteoblasts through the phosphorylation activation of p38/ERK MAP kinase signaling cascades *in vitro*. Irisin induces a rapid upregulation of Atf4 by stimulating the phosphorylation of MAPK ERK1 and ERK2 (pERK) (Storlino et al., 2020). Furthermore, as a downstream mediator of parathyroid hormone (PTH), Atf4 is involved in the promotion of PTH-mediated osteoblast/preosteoblast proliferation and the inhibition of osteoblast/osteocyte apoptosis (Kan et al., 2022). Similarly, Xue et al. (2022) demonstrated that irisin activates the αV integrin-induced ERK/STAT pathway, which in turn enhances BMP2 expression and activates the BMP/SMAD signaling cascade, ultimately promoting osteogenic differentiation. Despite these findings, the precise role of irisin in the p38/ERK MAPK signaling pathway remains insufficiently understood, warranting further research.

AMPK serves as a highly conserved regulator of cellular metabolism, playing an indispensable part in maintaining cellular homeostasis through energy regulation (Göransson et al., 2023). Research has shown that the activation of AMPK can inhibit inflammatory responses, promote chondrocyte proliferation, and prevent chondrocyte apoptosis. These signaling pathways confer chondroprotective effects (Feng et al., 2020), yet the exact regulatory mechanisms linking AMPK and irisin in bone metabolism remain to be fully explored (Ning et al., 2022). Tao et al. (2024) discovered that FNDC5/irisin binds to the scaffolding domain of caveolin-1 (Cav1) during endocytosis into osteoblasts, which then binds to AMPKα, forming a FNDC5/irisin-Cav1-AMPKα complex that activates the AMPK pathway. Nuclear factor E2-related factor 2 (Nrf2), a downstream target of the AMPK pathway, increases the transcription of HMOX1 and Fpn (Grignano et al., 2020; Soares and Hamza, 2016). This research also confirmed that irisin upregulates E2F2 in osteoblasts through an HMOX1-dependent mechanism, thereby promoting osteoblast proliferation by enhancing the S and G2/M phases of the cell cycle (Tao et al., 2024). This suggests that irisin can indirectly regulate the cell cycle through HMOX1, thereby promoting osteoblast proliferation. Additionally, Fpn upregulation in osteoblasts enhances iron removal, inhibiting osteoblast ferroptosis (Tao et al., 2024). Reduced ferroptosis, coupled with increased osteoblast proliferation, supports bone formation and prevents potential bone loss.

It is noteworthy that mixtures combining multiple cytokines may exhibit even greater therapeutic potential in OA. For instance, Recombinant human BMP-2 (rh-BMP-2), a critical growth factor widely applied in bone regeneration and fracture repair, demonstrates a remarkable synergistic effect when combined with irisin. Ohyama et al. have shown that combining rh-BMP-2 with recombinant irisin (re-irisin) significantly increases the mRNA expression levels of osteoblast differentiation markers (e.g., Runx2, ALP, OCN, and OPN) and enhances ectopic bone formation compared to the rh-BMP-2 alone group (Ohyama et al., 2024).

### 4.4 Irisin and osteoclasts

**FIGURE 4:**
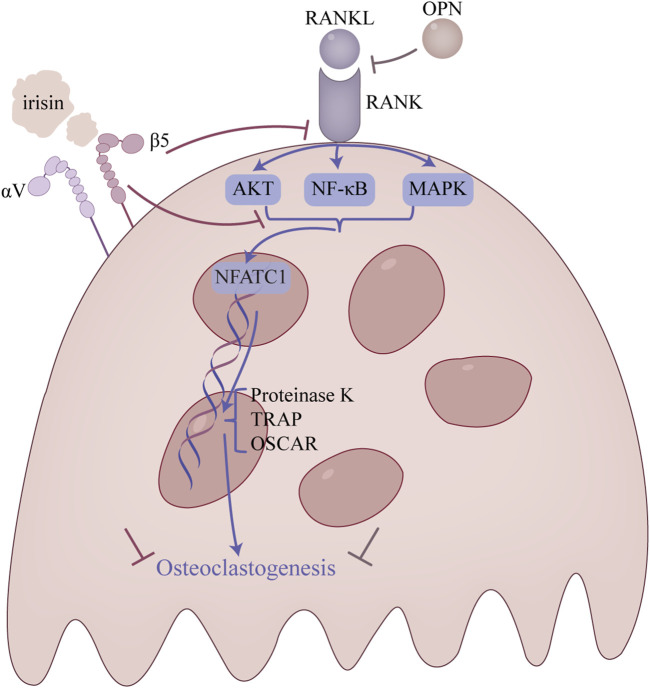
Main regulatory mechanism of irisin in osteoclasts. This figure is a summary of the main pathways involved in the attenuation of osteoclast overproduction.

Osteoclasts, multinucleated cells derived from hematopoietic precursors in bone marrow (Teitelbaum, 2000; Asagiri and Takayanagi, 2007; Boyle et al., 2003), are solely responsible for bone resorption (Yin et al., 2019). Over-activation of these cells perturbs bone metabolic balance, leading to enhanced resorption, reduced bone mass, and compromised microarchitecture (Teitelbaum, 2000; Jin et al., 2017). Important cytokines, including RANKL and macrophage colony-stimulating factor (M-CSF), are essential for regulating the proliferation and differentiation of osteoclast precursors. Notably, these cytokines are expressed by osteoblast lineage cells (Fumoto et al., 2014) or osteocytes (Xiong et al., 2011; Nakashima et al., 2011). Upon release, RANKL binds to its receptor RANK on osteoclast precursor cells, a process upregulated by M-CSF (Park et al., 2017). This interaction initiates signaling through the NF-κB and MAPK pathways, culminating in the activation of the transcription factor NFATc1 (Huang et al., 2006; Kim K. et al., 2018; Cui et al., 2020; Lee et al., 2016). Studies have shown that NFATc1 can directly promote the expression of osteoclast-specific markers, including TRAP, osteoclast-associated receptor (OSCAR), and cathepsin K (CTSK). Consequently, this process facilitates osteoclast differentiation and bone resorption (Bharti et al., 2004), ultimately promoting osteoclast formation. Irisin has been shown to inhibit bone resorption by downregulating these pathways, thereby suppressing osteoclast formation (Figure 4).

A crucial mechanism in the development of bone resorption is the NF-κB signaling pathway. The NF-κB signaling pathway is phosphorylated to cause the osteoclast to mature when the RANK on the surface of the osteoclast precursor cell attaches to RANKL (Enjuanes et al., 2010). The researchers found that irisin inhibits RANKL-induced degradation of IκBα and phosphorylation of p65, thereby suppressing the NF-κB signaling pathway and osteoclast differentiation (Sun et al., 1993; Ma et al., 2018b).

Moreover, the MAPK signaling pathway, which includes JNK, ERK, and p38 signaling pathways, serves as an important regulatory pathway in osteoclast formation (Stevenson et al., 2011). It was discovered that irisin altered the activation pattern of the RANKL-induced MAPK signaling pathway (Enjuanes et al., 2010). In particular, the p-ERK peak advanced while remaining high in response to irisin, while the p-JNK peak was dropped and delayed, and the p-p38 peak was delayed but increased. Additionally, irisin was shown to promote RAW264.7 cell proliferation by inducing activation of the p38 and JNK signaling pathway, as demonstrated in the presence of p38 and JNK inhibitors. However, it is noteworthy that the inhibition of osteoclast differentiation by irisin was not mediated by these pathways, suggesting that irisin may have a distinct effect on the MAPK pathway (Enjuanes et al., 2010).

Despite these findings, conflicting results regarding the effects of irisin on osteoclasts have emerged. Some studies suggest that irisin acts as a key factor in inducing bone remodeling. For example, Kim et al. demonstrated that by deactivating osteocytic osteolysis and osteoclastic bone resorption, 9-month-old ovariectomized FNDC5 global KO mice are protected against trabecular bone loss brought on by ovariectomy (Kim H. et al., 2018). In contract, Estell et al. found that bone marrow progenitor cells were dose-dependently stimulated to differentiate osteoclasts under treatment with different concentrations of recombinant irisin. Specifically, low concentrations of irisin had little effect on the number of osteoclasts *in vitro*, while higher concentrations of irisin reduced the number of osteoclasts (Estell et al., 2020). In this regard, Estell et al. (2020) suggested that the dose and duration of irisin exposure may be key determinants of skeletal response, drawing parallels to the bidirectional regulatory effects of PTH *in vivo*. Moreover, the use of exclusive single-sex mice in most studies may have been a factor in the conflicting results (Shimonty et al., 2024). Irisin triggers bone resorption in females by activating osteocytes and osteoclasts but protects against osteolysis in males during calcium deficiency, highlighting sex-specific skeletal effects (Shimonty et al., 2024).

### 4.5 Irisin and osteocytes

Osteocytes, comprising over 90% of skeletal cells, are integral to bone homeostasis. Their neuron-like dendritic processes traverse the bone’s tubules, facilitating communication within the mineralized matrix and with peripheral cells. This network regulates osteoblast-driven bone formation and osteoclast-mediated bone resorption, thereby having an impact on overall bone metabolism. Impairment of this dendritic network can be a cause of bone fragility (Lm and Sl, 2019). Podoplanin (PDPN), also known as E11/gp38, is a glycoprotein predominantly expressed in newly embedded osteoblasts. *In vitro* and *in vivo* studies have both demonstrated that PDPN is essential for the formation of dendrites and the development of a normal osteocytic canalicular network (Zhang et al., 2006; Staines et al., 2017). Storlino et al. (2020) have shown that irisin significantly increases the mRNA expression of PDPN in MLO-Y4 cells, implicating its role in promoting osteocyte dendrite formation. However, further studies are still necessary to determine if this effect occurs *in vivo*, which would support the hypothesis that irisin enhances cortical bone mass by improving the tubular network in osteocytes (Colaianni et al., 2021).

**FIGURE 5:**
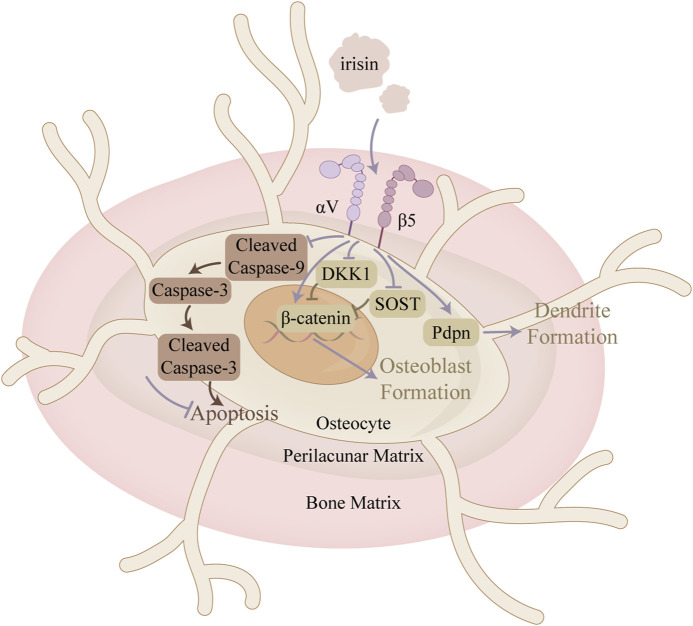
Mechanism of action of irisin in osteocytes. This figure illustrates the mechanism of irisin in restoring the balance between apoptosis and proliferation of bone cells in the context of OA. In addition, irisin promotes dendrite formation, thereby enhancing intercellular communication between osteocytes.

Irisin directly promotes osteocyte proliferation and inhibits apoptosis, suggesting a significant role in regulating bone metabolism and alleviating OA (Figure 5). Irisin mitigated the H_2_O_2_-induced decline in proliferative activity of MLO-Y4 by activating the MAPK signaling pathway and regulating BAX, Bcl-2, and c-myc expression (He et al., 2019). In mice induced with anterior cruciate ligament-transverse (ACLT), He et al. (2020) observed that irisin not only attenuated articular cartilage degeneration but also reduced the levels of apoptotic markers in the subchondral bone, including Caspase-3, BAX, and MMP-13. It was also suggested that irisin stimulated the LC3-II to LC3-I ratio, which serves as a critical marker for monitoring the initiation of autophagy, and the expression of LC3 and Ulk1 mRNA in MLO-Y4, indicating its role in reducing autophagy in osteocytes (Li et al., 2024). Furthermore, irisin improved the subchondral bone microstructure, which contributes to slowing the progression of OA. Bone microstructure parameters, including Tb. N and BV/TV, were also improved in the irisin-treated group (He et al., 2020).

Osteocytes can also indirectly regulate osteoblast differentiation and function by modulating Wnt/β-catenin signaling through the production of Wnt antagonists such as SOST and dickkopf-1 (Dkk1). Storlino et al. demonstrated that intermittent treatment with irisin significantly downregulated SOST expression in MLO-Y4 osteocytes, counteracting stretch-induced upregulation, while continuous exposure had no effect on that (Storlino et al., 2020). Conversely, Kim H. et al. (2018) observed an increase in SOST expression following six consecutive days of irisin treatment (10 nM). These findings indicate that the mode of irisin exposure is crucial. Regarding Dkk1, Storlino et al. provided *in vitro* evidence that irisin also directly affects Dkk1 expression, an inhibitor of the Wnt signaling pathway, in osteocytes (Storlino et al., 2020).

## 5 Cartilage metabolism

**FIGURE 6:**
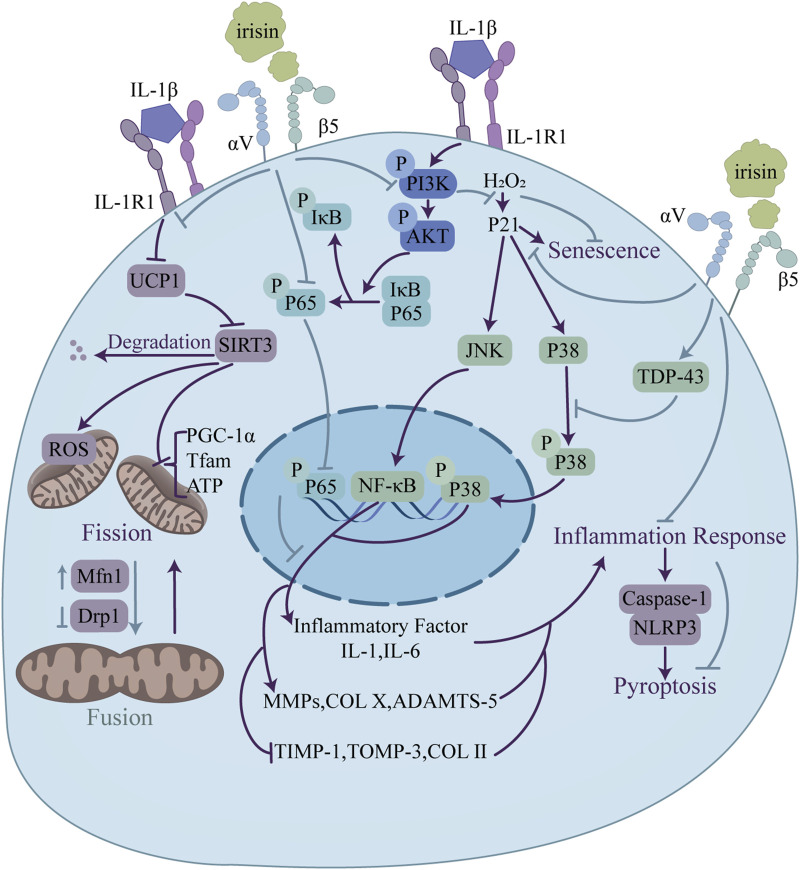
Regulatory functions of irisin in osteoarthritis relief through modulation of chondrocyte metabolism. Irisin enhances mitochondrial function via UCP1/SIRT3 signaling, upregulating Mfn1 and downregulating Drp1 to promote mitochondrial fusion. Irisin also inhibits pyroptosis through the NLRP3/caspase-1 pathway. Additionally, irisin blocks the PI3K/Akt/NF-κB signaling and suppresses JNK and p38 MAPK. This regulation leads to a balance in ECM homeostasis, with upregulation of genes promoting ECM formation (TIMP-1, COLII) and downregulation of genes linked to ECM degradation (MMP-1, COL X).

Irisin displays a differential expression pattern during chondrogenesis, suggesting its potential involvement in regulating this process (Li X. et al., 2021). Inflammatory state in OA significantly reduces the expression and total level of Irisin/FNDC5 in human and mouse cartilage tissues (Wang et al., 2020; Li X. et al., 2021), as well as in serum (Zhu et al., 2021), and leads to an increase in chondrocyte apoptosis (Wang et al., 2020). These findings establish a foundation for further investigation into the potential of irisin to promote chondrocyte survival and enhance cartilage integrity. Numerous *in vivo* and *in vitro* studies have demonstrated that irisin functions in cartilage metabolism through the regulation of various signaling pathways essential for chondrocyte proliferation, senescence, apoptosis, oxidative stress, and ECM synthesis and degradation, which ultimately improves OA progress and relieves OA syndromes (Figure 6) (Table 3).

**TABLE 3:** Role of irisin in cartilage metabolism during osteoarthritis progression.

Chondrocyte type	Dosage	Effects	References
Chondrocytes isolated from the knee articular cartilage of 4-week-old SD rats	0, 5, 10 ng/mL; 1 h	Inhibition of PI3K/Akt/NF-κB signaling pathway	Jia et al. (2022)
		Inhibited activity of NLRP3/caspase-1	
Chondrocytes isolated from he hips, femurs and tibiae of 7-day-old mice	1, 5 and 10 ng/mL; 6 h	Upregulated autophagy and apoptosis	Wang et al. (2020)
		Reversed Sirt3 and UCP-1 pathways	
		Improved mitochondrial function and ECM components synthesis	
human osteoarthritic chondrocytes (hOAC)	25 ng/mL; 14 days	Inhibition of p38, Akt, JNK, and NF-κB signaling pathways	Vadalà et al. (2020)
		Increased cell proliferation and ECM component synthesis	
		Inhibited ECM degradation and inflammatory factor expression	

Abbreviations: PI3K, phosphatidylinositol 3-kinase; NF-κB, nuclear factor kappa-B; NLRP3, NOD-like receptor thermal protein domain associated protein 3; Sirt3, Sirtuin 3; UCP-1, uncoupling protein 1; ECM, extracellular matrix; hOAC, human osteoarthritic chondrocytes; p38, p38 mitogen-activated protein kinase; JNK, c-Jun N-terminal kinase.

### 5.1 Irisin and chondrocytes

Excessive chondrocyte apoptosis and diminished proliferation are hallmarks of cartilage degradation. The PI3K/Akt pathway, a key modulator of chondrocyte survival and apoptosis, can mitigate osteoarthritis by curbing chondrocyte death (Sun et al., 2020). According to current research, findings regarding the PI3K/Akt signaling pathway are largely consistent. Jia et al. (2022) demonstrated that irisin ameliorates IL-1β-induced pyroptosis in chondrocytes by inhibiting the inflammation-induced NLRP3/caspase-1 pathway. The work also revealed that irisin alleviates chondrocyte inflammation by blocking IL-1β-induced NF-κB p65 nuclear translocation and suppressing the PI3K/Akt/NF-κB signaling pathway, as evidenced by the downregulation of PI3K, Akt and NF-κB p65.

OA usually accompanies an inflammatory milieu that accelerates chondrocyte demise and cartilage breakdown (Arra et al., 2020). Studies have implicated various signaling pathways in OA pathogenesis, with MAPK signaling pathway being a crucial player. Dysregulation of this pathway can exacerbate inflammatory responses, resulting in the release of substantial quantities of massive cartilage matrix degrading enzymes and the intensification of cartilage degeneration (Li et al., 2022). In terms of the MAPK signaling pathway, Zhang et al. (2021) confirmed that irisin promotes the expression of TDP-43 in cartilage tissue, inhibiting the activation and nuclear translocation of p38 MAPK, which in turn suppresses the expression of downstream inflammatory cytokines such as IL-1β and IL-6, thus alleviating OA. On the other hand, the regulation of irisin on the ERK signaling pathway displays controversial results. Posa et al. (2023) found that irisin enhances the expression of pERK in HAC cells, while Vadalà (Vadalà et al., 2020) reported that irisin has no significant effect on the level of pERK protein in hOACs. The difference in response of pERK to irisin may be related to its dosage and treatment time. Vadalà’s study (Vadalà et al., 2020) also confirmed that irisin restores the normal ECM gene expression profile in hOACs by inhibiting the p38, Akt, JNK, and NF-κB signaling pathways, downregulating IL-1, IL-6, MMP-1, MMP-13, iNOS, and COL X, while upregulating TIMP-1, TIMP-3, and COL II levels.

In addition, the study by Wang et al. (2020) provides a fresh perspective on investigating the mechanisms behind chondrocyte fate and metabolism. It focused on the effects of irisin in improving inflammatory chondrocyte survival and ECM anabolism by ameliorating mitochondrial dysfunction with defective mitochondrial autophagy and preserving mitochondrial activity. They revealed, and elucidated the mechanism behind this by finding reduced levels of FNDC5 and LC3-II expression and increased levels of oxidative DNA damage marker 8-hydroxydeoxyguanosine (8-OHdG) and apoptosis in human osteoarthritic articular chondrocytes. Irisin treatment reversed the disruption of the dynamic balance between mitochondrial fusion and division by IL-1β, and improved mitochondrial autophagic vesicle development and mitochondrial formation, which were achieved by upregulation of the mitochondrial fusion marker, Mfn1, downregulation of the mitochondrial division marker, Drp1, and by increased expression of mitochondrial autophagy-associated proteins, PINK1 and Parkin. Treatment with irisin reversed the effects of IL -1β inhibition of Sirt3 and UCP-1 signaling pathways, which play key roles in maintaining mitochondrial integrity and biosynthesis, significantly ameliorated PGC-1α, Tfam loss, and deficient ATP production in inflammatory chondrocytes to maintain their membrane potential. In addition, Irisin inhibited IL-1β-mediated apoptosis and autophagy defects in chondrocytes by improving the expression of Atg4, Atg12, and p62, and by promoting LC3-II conversion.

### 5.2 Irisin and the extracellular matrix of articular cartilage

Irisin exerts multiple beneficial effects on the extracellular matrix (ECM), including promoting matrix production, inhibiting degradation, enhancing chondrocyte differentiation and proliferation, and reducing cell death. At the level of gene regulation, irisin effectively promoted chondrocyte differentiation and tissue growth by decreasing SOX9 expression (Wang et al., 2020; Posa et al., 2023). Inhibition of COX2 and iNOS gene expression by Irisin contributed to ECM protection by reducing oxidative stress at the cellular level (Li et al., 2020). For ECM components, Irisin dose-dependently promoted ECM production in inflammatory chondrocytes, reversing IL-1β-induced degradation of collagen II (Wang et al., 2020), proteoglycans (Wang et al., 2020; Posa et al., 2023), and glycosaminoglycans (GAGs) (Zhang et al., 2021; Vadalà et al., 2000). Additionally, Irisin inhibited ECM degradation by significantly downregulating the expression of MMPs, including MMP-3 (Chen et al., 2022), MMP-9 (Wang et al., 2020), MMP-13 (Chen et al., 2022), and metalloprotease ADAMTS-5 (Posa et al., 2023), thereby preventing ECM breakdown. Irisin also reduced inflammatory mediators such as IL-6 (Chen et al., 2022), IL-1β, and TNF-α (Posa et al., 2023), which is conducive to maintaining a stable ECM environment.

## 6 Conclusion and perspective

Irisin, a myokine produced during exercise by cleavage of the membrane protein FNDC5, has gained increasing attention with advancements in genetic editing techniques. Recent studies using FNDC5 KO (Li et al., 2021; Giorgino et al., 2023), gene overexpression (Li et al., 2021; Liu et al., 2022), and transgenic models (Estell et al., 2020) have validated the role of irisin on bone metabolism. Given the significance of the FNDC5 gene in OA and the sparse literature on its comprehensive role, this review emphasizes its exercise-induced effects and regulatory impact on skeletal health.

Bone and cartilage metabolism are closely interconnected during the complex progression of OA. Compared to bone metabolism, fewer reviews have explored the potential role and regulatory effects of irisin on chondrocytes. This review aims to provide a broader perspective by investigating the multifaceted signaling pathways that irisin may modulate to influence bone and cartilage homeostasis, thereby revealing its potential in mitigating OA progression and associated symptoms. Here, we review *in vitro* and *in vivo* evidence to clarify the multifaceted effects of irisin on chondrocyte function, ECM dynamics, inflammatory mediators, cartilage matrix histology, and bone metabolism.

Although we explore the connection between the biological effects of irisin on bone and cartilage homeostasis and OA development, some important recent literature was not included due to limited research on specific areas. Current research on irisin and OA has primarily focused on its effects on end-stage knee OA. Future studies could investigate the early-stage changes in irisin levels and its mechanisms of action in preserving cartilage and subchondral bone integrity, as well as refining studies of OA in other body sites. Moreover, examining how irisin mitigates organelle dysfunction to maintain skeletal cell survival and function in OA is a promising area for future research (Wang et al., 2020).

These insights deepen the current understanding of the biological functions of irisin and offer promising directions for developing novel OA therapies. We highlight the promising potential of irisin as a therapeutic agent for OA and delve into its mechanisms in alleviating OA symptoms generally. However, in a study examining the association of synovial irisin level with progression of KOA determined by the Kellgren-Lawrence (KL) criteria, irisin levels varied significantly among patient groups of different KOA inflammatory phenotypes (KOIP), with distinct patterns observed in relation to radiographic progression status (Calvet et al., 2024). Therefore, it is imperative to conduct comparative studies to elucidate the mitigating effects of irisin across distinct subtypes of OA. Studies have shown that irisin levels are significantly lower in obese individuals, which may be associated with metabolic dysfunction (Wang et al., 2022). In individuals with obesity and type 2 diabetes mellitus (T2DM), low levels of irisin are associated with the progression of OA (Kurdiova et al., 2014; Liu et al., 2013). Obesity impacts joint health through increased mechanical load and metabolic/inflammatory changes. Irisin deficiency impairs the protective effects on bone metabolism, accelerating OA progression (Colaianni et al., 2015). In T2DM, irisin enhances muscle insulin signaling and glucose uptake, indirectly supporting bone health. Irisin also interacts with adipocytokines such as leptin and adiponectin, increasing adiponectin levels to improve insulin sensitivity and reduce inflammation. Metabolic hormone dysregulation and non-esterified fatty acid (NEFA) release in T2DM affect bone metabolism, where irisin may play a beneficial role (Zhang et al., 2014). Metabolic disorders in obesity and diabetes can influence irisin levels, exacerbating OA pathology. Thus, irisin serves as a protective factor at the intersection of obesity, diabetes, and OA, with its level changes closely related to disease progression (Kurdiova et al., 2014). Further research is needed to systematically compare the efficacy of irisin with current OA treatments, evaluate its anti-inflammatory effects, and ensure its safety. Such investigations will enhance our understanding of the clinical potential of irisin for OA treatment and may provide a solid theoretical foundation for developing innovative and targeted strategies to alleviate the progression of OA.

## References

[B1] Arra M. Swarnkar G. Ke K. Otero J. E. Ying J. Duan X. (2020). LDHA-mediated ROS generation in chondrocytes is a potential therapeutic target for osteoarthritis. Nat. Commun. 11 (1), 3427. 10.1038/s41467-020-17242-0 32647171 PMC7347613

[B2] Asagiri M. Takayanagi H. (2007). The molecular understanding of osteoclast differentiation. Bone 40 (2), 251–264. 10.1016/j.bone.2006.09.023 17098490

[B3] Bharti A. C. Takada Y. Shishodia S. Aggarwal B. B. (2004). Evidence that receptor activator of nuclear factor (NF)-kappaB ligand can suppress cell proliferation and induce apoptosis through activation of a NF-kappaB-independent and TRAF6-dependent mechanism. J. Biol. Chem. 279 (7), 6065–6076. 10.1074/jbc.M308062200 14645259

[B4] Boström P. Wu J. Jedrychowski M. P. Korde A. Ye L. Lo J. C. (2012). A PGC1-α-dependent myokine that drives brown-fat-like development of white fat and thermogenesis. Nature 481 (7382), 463–468. 10.1038/nature10777 22237023 PMC3522098

[B5] Boyle W. J. Simonet W. S. Lacey D. L. (2003). Osteoclast differentiation and activation. Nature 423 (6937), 337–342. 10.1038/nature01658 12748652

[B6] Calvet J. Berenguer-Llergo A. Orellana C. García-Manrique M. Rusiñol M. Garcia-Cirera S. (2024). Specific-cytokine associations with outcomes in knee osteoarthritis subgroups: breaking down disease heterogeneity with phenotyping. Arthritis Res. Ther. 26 (1), 19. 10.1186/s13075-023-03244-y 38212829 PMC10782658

[B7] Cao F. Xu Z. Li X. X. Fu Z. Y. Han R. Y. Zhang J. L. (2024). Trends and cross-country inequalities in the global burden of osteoarthritis, 1990–2019: a population-based study. Ageing Res. Rev. 99, 102382. 10.1016/j.arr.2024.102382 38917934

[B8] Chen T. Peng Y. Hu W. Shi H. Li P. Que Y. (2022). Irisin enhances chondrogenic differentiation of human mesenchymal stem cells via Rap1/PI3K/AKT axis. Stem cell Res. and Ther. 13 (1), 392. 10.1186/s13287-022-03092-8 35922833 PMC9351134

[B9] Chen X. Sun K. Zhao S. Geng T. Fan X. Sun S. (2020). Irisin promotes osteogenic differentiation of bone marrow mesenchymal stem cells by activating autophagy via the Wnt//β-catenin signal pathway. Cytokine 136, 155292. 10.1016/j.cyto.2020.155292 32950809

[B10] Colaianni G. Cuscito C. Mongelli T. Pignataro P. Buccoliero C. Liu P. (2015). The myokine irisin increases cortical bone mass. Proc. Natl. Acad. Sci. U. S. A. 112 (39), 12157–12162. 10.1073/pnas.1516622112 26374841 PMC4593131

[B11] Colaianni G. Errede M. Sanesi L. Notarnicola A. Celi M. Zerlotin R. (2021). Irisin correlates positively with BMD in a cohort of older adult patients and downregulates the senescent marker p21 in osteoblasts. J. bone mineral Res. official J. Am. Soc. Bone Mineral Res. 36 (2), 305–314. 10.1002/jbmr.4192 33053231

[B12] Cui J. Li X. Wang S. Su Y. Chen X. Cao L. (2020). Triptolide prevents bone loss via suppressing osteoclastogenesis through inhibiting PI3K-AKT-NFATc1 pathway. J. Cell. Mol. Med. 24 (11), 6149–6161. 10.1111/jcmm.15229 32347017 PMC7294126

[B13] Di M. G. Alessio N. Ambrosino A. Al Sammarraie S. H. A. Monda M. Di Bernardo G. (2024). Irisin influences the *in vitro* differentiation of human mesenchymal stromal cells, promoting a tendency toward beiging adipogenesis. J. Cell. Biochem. 125 (5), e30565. 10.1002/jcb.30565 38591469

[B14] Enjuanes A. Ruiz-Gaspà S. Peris P. Ozalla D. Álvarez L. Combalia A. (2010). The effect of the alendronate on OPG/RANKL system in differentiated primary human osteoblasts. Endocrine 37 (1), 180–186. 10.1007/s12020-009-9285-9 20963568

[B15] Estell E. G. Le P. T. Vegting Y. Kim H. Wrann C. Bouxsein M. L. (2020). Irisin directly stimulates osteoclastogenesis and bone resorption *in vitro* and *in vivo* . eLife 9, e58172. 10.7554/eLife.58172 32780016 PMC7444909

[B16] Feng X. McDonald J. M. (2011). Disorders of bone remodeling. Annu. Rev. Pathol. 6, 121–145. 10.1146/annurev-pathol-011110-130203 20936937 PMC3571087

[B17] Feng X. Pan J. Li J. Zeng C. Qi W. Shao Y. (2020). Metformin attenuates cartilage degeneration in an experimental osteoarthritis model by regulating AMPK/mTOR. Aging (Albany NY) 12 (2), 1087–1103. 10.18632/aging.102635 31945013 PMC7053618

[B18] Fumoto T. Takeshita S. Ito M. Ikeda K. (2014). Physiological functions of osteoblast lineage and T cell-derived RANKL in bone homeostasis. J. Bone Min. Res. 29 (4), 830–842. 10.1002/jbmr.2096 24014480

[B19] Giorgino R. Albano D. Fusco S. Peretti G. M. Mangiavini L. Messina C. (2023). Knee osteoarthritis: epidemiology, pathogenesis, and mesenchymal stem cells: what else is new? An update. Int. J. Mol. Sci. 24 (7), 6405. 10.3390/ijms24076405 37047377 PMC10094836

[B20] Goldring M. B. Birkhead J. Sandell L. J. Kimura T. Krane S. M. (1988). Interleukin 1 suppresses expression of cartilage-specific types II and IX collagens and increases types I and III collagens in human chondrocytes. J. Clin. Invest 82 (6), 2026–2037. 10.1172/JCI113823 3264290 PMC442785

[B21] Göransson O. Kopietz F. Rider M. H. (2023). Metabolic control by AMPK in white adipose tissue. Trends Endocrinol. and Metabolism 34 (11), 704–717. 10.1016/j.tem.2023.08.011 37673765

[B22] Grignano E. Birsen R. Chapuis N. Bouscary D. (2020). From iron chelation to overload as a therapeutic strategy to induce ferroptosis in leukemic cells. Front. Oncol. 10, 586530. 10.3389/fonc.2020.586530 33042852 PMC7530268

[B23] He Z. Li H. Han X. Zhou F. Du J. Yang Y. (2020). Irisin inhibits osteocyte apoptosis by activating the Erk signaling pathway *in vitro* and attenuates ALCT-induced osteoarthritis in mice. Bone 141, 115573. 10.1016/j.bone.2020.115573 32768686

[B24] He Z. Li H. Zhou F. Han X. Chu L. Zhang S. (2019). Irisin attenuates osteoarthritis by inhibiting apoptosis of osteocytes through activating erk signaling pathway. Osteoarthr. Cartil. 27, S194. 10.1016/j.joca.2019.02.298

[B25] Hu Y. Chen X. Wang S. Jing Y. Su J. (2021). Subchondral bone microenvironment in osteoarthritis and pain. Bone Res. 9 (1), 20. 10.1038/s41413-021-00147-z 33731688 PMC7969608

[B26] Huang H. Ryu J. Ha J. Chang E. J. Kim H. J. Kim H. M. (2006). Osteoclast differentiation requires TAK1 and MKK6 for NFATc1 induction and NF-kappaB transactivation by RANKL. Cell Death Differ. 13 (11), 1879–1891. 10.1038/sj.cdd.4401882 16498455

[B27] Huh J. Y. Mougios V. Kabasakalis A. Fatouros I. Siopi A. Douroudos I. I. (2014). Exercise-induced irisin secretion is independent of age or fitness level and increased irisin may directly modulate muscle metabolism through AMPK activation. J. Clin. Endocrinol. Metab. 99 (11), E2154–E2161. 10.1210/jc.2014-1437 25119310

[B28] Hunter D. J. Bierma-Zeinstra S. (2019). Osteoarthritis. Lancet 393 (10182), 1745–1759. 10.1016/S0140-6736(19)30417-9 31034380

[B29] Jedrychowski M. P. Wrann C. D. Paulo J. A. Gerber K. K. Szpyt J. Robinson M. M. (2015). Detection and quantitation of circulating human irisin by tandem mass spectrometry. Cell Metab. 22 (4), 734–740. 10.1016/j.cmet.2015.08.001 26278051 PMC4802359

[B30] Jia S. Yang Y. Bai Y. Wei Y. Zhang H. Tian Y. (2022). Mechanical stimulation protects against chondrocyte pyroptosis through irisin-induced suppression of PI3K/Akt/NF-κB signal pathway in osteoarthritis. Front. cell Dev. Biol. 10, 797855. 10.3389/fcell.2022.797855 35356271 PMC8959944

[B31] Jin Y. X. Wu P. Mao Y. F. Wang B. Zhang J. F. Chen W. L. (2017). Chinese herbal medicine for osteoporosis: a meta-analysis of randomized controlled trials. J. Clin. Densitom. 20 (4), 516–525. 10.1016/j.jocd.2017.07.003 28893468

[B32] Kan T. He Z. Du J. Xu M. Cui J. Han X. (2022). Irisin promotes fracture healing by improving osteogenesis and angiogenesis. J. Orthop. Transl. 37, 37–45. 10.1016/j.jot.2022.07.006 PMC9513699 36196152

[B33] Kawao N. Moritake A. Tatsumi K. Kaji H. (2018). Roles of irisin in the linkage from muscle to bone during mechanical unloading in mice. Calcif. tissue Int. 103 (1), 24–34. 10.1007/s00223-018-0387-3 29332162

[B34] Kim H. Wrann C. D. Jedrychowski M. Vidoni S. Kitase Y. Nagano K. (2018). Irisin mediates effects on bone and fat via αV integrin receptors. Cell 175 (7), 1756–1768. 10.1016/j.cell.2018.10.025 30550785 PMC6298040

[B35] Kim K. Kim T. H. Ihn H. J. Kim J. E. Choi J. Y. Shin H. I. (2018). Inhibitory effect of purpurogallin on osteoclast differentiation *in vitro* through the downregulation of c-fos and NFATc1. Int. J. Mol. Sci. 19 (2), 601. 10.3390/ijms19020601 29463002 PMC5855823

[B36] Kong H. Wang X. Q. Zhang X. A. (2022). Exercise for osteoarthritis: a literature review of pathology and mechanism. Front. Aging Neurosci. 14, 854026. 10.3389/fnagi.2022.854026 35592699 PMC9110817

[B37] Kourí J. B. Rosales-Encína J. L. Chaudhuri P. Luna J. Mena R. (1997). Apoptosis in human osteoarthritic cartilage: a microscopy report. Med. Sci. Res. 25, 245–248.

[B38] Kurdiova T. Balaz M. Vician M. Maderova D. Vlcek M. Valkovic L. (2014). Effects of obesity, diabetes and exercise on Fndc5 gene expression and irisin release in human skeletal muscle and adipose tissue: *in vivo* and *in vitro* studies. J. Physiol. 592 (5), 1091–1107. 10.1113/jphysiol.2013.264655 24297848 PMC3948565

[B39] Lee K. Chung Y. H. Ahn H. Kim H. Rho J. Jeong D. (2016). Selective regulation of MAPK signaling mediates RANKL-dependent osteoclast differentiation. Int. J. Biol. Sci. 12 (2), 235–245. 10.7150/ijbs.13814 26884720 PMC4737679

[B40] Li H. Luo D. Xie W. Ye W. Chen J. Alberton P. (2024). Irisin reduces senile osteoporosis by inducing osteocyte mitophagy through Ampk activation. ISCIENCE 27 (11), 111042. 10.1016/j.isci.2024.111042 39559753 PMC11570468

[B41] Li H. Qin S. Liang Q. Xi Y. Bo W. Cai M. (2021a). Exercise training enhances myocardial mitophagy and improves cardiac function via irisin/FNDC5-PINK1/parkin pathway in MI mice. Biomedicines 9 (6), 701. 10.3390/biomedicines9060701 34205641 PMC8234442

[B42] Li X. Liu Y. Liu Q. Wang S. Ma Y. Jin Q. (2020). Recombinant human irisin regulated collagen II, matrix metalloproteinase-13 and the Wnt/β-catenin and NF-κB signaling pathways in interleukin-1β-induced human SW1353 cells. Exp. Ther. Med. 19 (4), 2879–2886. 10.3892/etm.2020.8562 32256772 PMC7086223

[B43] Li X. Zhu X. Wu H. Van Dyke T. E. Xu X. Morgan E. F. (2021b). Roles and mechanisms of irisin in attenuating pathological features of osteoarthritis. Front. Cell Dev. Biol. 9, 703670. 10.3389/fcell.2021.703670 34650969 PMC8509718

[B44] Li Z. Dai A. Yang M. Chen S. Deng Z. Li L. (2022). p38MAPK signaling pathway in osteoarthritis: pathological and therapeutic aspects. JIR 15, 723–734. 10.2147/JIR.S348491 PMC8820459 35140502

[B45] Liu A. Chen Y. Zhong D. Wang C. Yu M. Liu C. (2022). CircRNA AFF4 induced by KDM1A promotes osteogenic differentiation through FNDC5/Irisin pathway. Mol. Med. 28 (1), 134. 10.1186/s10020-022-00557-7 36401176 PMC9673395

[B46] Liu C. Liu A. S. Zhong D. Wang C. G. Yu M. Zhang H. W. (2021b). Circular RNA AFF4 modulates osteogenic differentiation in BM-MSCs by activating SMAD1/5 pathway through miR-135a-5p/FNDC5/Irisin axis. Cell Death and Dis. 12 (7), 631. 10.1038/s41419-021-03877-4 PMC8213698 34145212

[B47] Liu J. J. Wong M. D. S. Toy W. C. Tan C. S. H. Liu S. Ng X. W. (2013). Lower circulating irisin is associated with type 2 diabetes mellitus. J. Diabetes Complicat. 27 (4), 365–369. 10.1016/j.jdiacomp.2013.03.002 23619195

[B48] Liu L. Guo J. Chen X. Tong X. Xu J. Zou J. (2021a). The role of irisin in exercise-mediated bone health. Front. cell Dev. Biol. 9, 668759. 10.3389/fcell.2021.668759 34017836 PMC8129548

[B49] Liu S. S. Zhou P. Zhang Y. (2016). Abnormal expression of key genes and proteins in the canonical Wnt/β-catenin pathway of articular cartilage in a rat model of exercise-induced osteoarthritis. Mol. Med. Rep. 13 (3), 1999–2006. 10.3892/mmr.2016.4798 26794964 PMC4768959

[B50] Lm T. L. Sl D. (2019). Changes in the osteocyte lacunocanalicular network with aging. Bone 122, 101–113. 10.1016/j.bone.2019.01.025 30743014 PMC6638547

[B51] Luo Y. Qiao X. Ma Y. Deng H. Xu C. C. Xu L. (2020). Disordered metabolism in mice lacking irisin. Sci. Rep. 10 (1), 17368. 10.1038/s41598-020-74588-7 33060792 PMC7567109

[B52] Ma Y. Qi M. An Y. Zhang L. Yang R. Doro D. H. (2018a). Autophagy controls mesenchymal stem cell properties and senescence during bone aging. Aging Cell 17 (1), e12709. 10.1111/acel.12709 29210174 PMC5770781

[B53] Ma Y. Qiao X. Zeng R. Cheng R. Zhang J. Luo Y. (2018b). Irisin promotes proliferation but inhibits differentiation in osteoclast precursor cells. FASEB J. 32 (11), 5813–5823. 10.1096/fj.201700983rr 29771602

[B54] Mao Y. Xu W. Xie Z. Dong Q. (2016). Association of irisin and CRP levels with the radiographic severity of knee osteoarthritis. Genet. Test. Mol. Biomarkers 20 (2), 86–89. 10.1089/gtmb.2015.0170 26625129

[B55] Metzger C. E. Anand N. S. Phan P. H. Bloomfield S. A. (2020). Hindlimb unloading causes regional loading-dependent changes in osteocyte inflammatory cytokines that are modulated by exogenous irisin treatment. NPJ Microgravity 6, 28. 10.1038/s41526-020-00118-4 33083525 PMC7542171

[B56] Nakashima T. Hayashi M. Fukunaga T. Kurata K. Oh-Hora M. Feng J. Q. (2011). Evidence for osteocyte regulation of bone homeostasis through RANKL expression. Nat. Med. 17 (10), 1231–1234. 10.1038/nm.2452 21909105

[B57] Ning K. Wang Z. Zhang X. A. (2022). Exercise-induced modulation of myokine irisin in bone and cartilage tissue-Positive effects on osteoarthritis: a narrative review. Front. Aging Neurosci. 14, 934406. 10.3389/fnagi.2022.934406 36062149 PMC9439853

[B58] Ohyama Y. Ohta Y. Sugama R. Minoda Y. Masuda S. Terai H. (2024). Effect of recombinant irisin on recombinant human bone morphogenetic protein-2 induced osteogenesis and osteoblast differentiation. Biochem. Biophys. Res. Commun. 734, 150787. 10.1016/j.bbrc.2024.150787 39368373

[B59] Park J. H. Lee N. K. Lee S. Y. (2017). Current understanding of RANK signaling in osteoclast differentiation and maturation. Mol. Cells 40 (10), 706–713. 10.14348/molcells.2017.0225 29047262 PMC5682248

[B60] Pekkala S. Wiklund P. K. Hulmi J. J. Ahtiainen J. P. Horttanainen M. Pöllänen E. (2013). Are skeletal muscle FNDC5 gene expression and irisin release regulated by exercise and related to health? J. Physiol. 591 (21), 5393–5400. 10.1113/jphysiol.2013.263707 24000180 PMC3936375

[B61] Posa F. Zerlotin R. Ariano A. Cosola M. D. Colaianni G. Fazio A. D. (2023). Irisin role in chondrocyte 3D culture differentiation and its possible applications. Pharmaceutics 15 (2), 585. 10.3390/pharmaceutics15020585 36839906 PMC9961836

[B62] Qiao X. Yong Qiao X. Nie Y. Ma Y. Xian Ma Y. Chen Y. (2016). Irisin promotes osteoblast proliferation and differentiation via activating the MAP kinase signaling pathways. Sci. Rep. 6, 18732. 10.1038/srep18732 26738434 PMC4704023

[B63] Richardson D. W. Dodge G. R. (2000). Effects of interleukin-1beta and tumor necrosis factor-alpha on expression of matrix-related genes by cultured equine articular chondrocytes. Am. J. Vet. Res. 61 (6), 624–630. 10.2460/ajvr.2000.61.624 10850836

[B64] Rojas-Ortega M. Cruz R. Vega-López M. A. Cabrera-González M. Hernández-Hernández J. M. Lavalle-Montalvo C. (2015). Exercise modulates the expression of IL-1β and IL-10 in the articular cartilage of normal and osteoarthritis-induced rats. Pathol. Res. Pract. 211 (6), 435–443. 10.1016/j.prp.2015.01.008 25702530

[B65] Shimonty A. Pin F. Prideaux M. Peng G. Huot J. Kim H. (2024). Deletion of FNDC5/irisin modifies murine osteocyte function in a sex-specific manner. eLife 12. 10.7554/eLife.92263 PMC11045224 38661340

[B66] Soares M. P. Hamza I. (2016). Macrophages and iron metabolism. Immunity 44 (3), 492–504. 10.1016/j.immuni.2016.02.016 26982356 PMC4794998

[B67] Staines K. A. Javaheri B. Hohenstein P. Fleming R. Ikpegbu E. Unger E. (2017). Hypomorphic conditional deletion of E11/Podoplanin reveals a role in osteocyte dendrite elongation. J. Cell Physiol. 232 (11), 3006–3019. 10.1002/jcp.25999 28488815 PMC5575468

[B68] Stevenson D. A. Schwarz E. L. Carey J. C. Viskochil D. H. Hanson H. Bauer S. (2011). Bone resorption in syndromes of the Ras/MAPK pathway. Clin. Genet. 80 (6), 566–573. 10.1111/j.1399-0004.2010.01619.x 21204800 PMC3246507

[B69] Storlino G. Colaianni G. Sanesi L. Lippo L. Brunetti G. Errede M. (2020). Irisin prevents disuse-induced osteocyte apoptosis. J. bone mineral Res. official J. Am. Soc. Bone Mineral Res. 35 (4), 766–775. 10.1002/jbmr.3944 31826311

[B70] Sun K. Luo J. Guo J. Yao X. Jing X. Guo F. (2020). The PI3K/AKT/mTOR signaling pathway in osteoarthritis: a narrative review. Osteoarthr. Cartil. 28 (4), 400–409. 10.1016/j.joca.2020.02.027 32081707

[B71] Sun S. C. Ganchi P. A. Ballard D. W. Greene W. C. (1993). NF-kappa B controls expression of inhibitor I kappa B alpha: evidence for an inducible autoregulatory pathway. Science 259 (5103), 1912–1915. 10.1126/science.8096091 8096091

[B72] Tao L. Wang J. Wang K. Liu Q. Li H. Xu S. (2024). Exerkine FNDC5/irisin-enriched exosomes promote proliferation and inhibit ferroptosis of osteoblasts through interaction with Caveolin-1. Aging Cell 23, e14181. 10.1111/acel.14181 38689463 PMC11320359

[B73] Teitelbaum S. L. (2000). Bone resorption by osteoclasts. Science 289 (5484), 1504–1508. 10.1126/science.289.5484.1504 10968780

[B74] Vadalà G. Di Giacomo G. Ambrosio L. Cannata F. Cicione C. Papalia R. (2020). Irisin recovers osteoarthritic chondrocytes *in vitro* . Cells 9 (6), 1478. 10.3390/cells9061478 32560375 PMC7348865

[B75] Wang F. S. Kuo C. W. Ko J. Y. Chen Y. S. Wang S. Y. Ke H. J. (2020). Irisin mitigates oxidative stress, chondrocyte dysfunction and osteoarthritis development through regulating mitochondrial integrity and autophagy. Antioxidants (Basel). 9 (9), 810. 10.3390/antiox9090810 32882839 PMC7555738

[B89] Wang X. Hu T. Ruan Y. Yao J. Shen H. Xu Y. (2022). The association of serum irisin with bone mineral density and turnover markers in new-onset type 2 diabetic patients. International Journal of Endocrinology 2022 1, 7808393. 35265126 10.1155/2022/7808393 PMC8901306

[B76] Xing S. Ma Y. Song B. Bai M. Wang K. Song W. (2025). Irisin reshapes bone metabolic homeostasis to delay age-related osteoporosis by regulating the multipotent differentiation of BMSCs via Wnt pathway. Front. Mol. Biosci. 11, 1524978. 10.3389/fmolb.2024.1524978 39840074 PMC11746060

[B77] Xiong J. Onal M. Jilka R. L. Weinstein R. S. Manolagas S. C. O’Brien C. A. (2011). Matrix-embedded cells control osteoclast formation. Nat. Med. 17 (10), 1235–1241. 10.1038/nm.2448 21909103 PMC3192296

[B78] Xue Y. Hu S. Chen C. He J. Sun J. Jin Y. (2022). Myokine Irisin promotes osteogenesis by activating BMP/SMAD signaling via αV integrin and regulates bone mass in mice. Int. J. Biol. Sci. 18 (2), 572–584. 10.7150/ijbs.63505 35002510 PMC8741853

[B79] Yao Q. Wu X. Tao C. Gong W. Chen M. Qu M. (2023). Osteoarthritis: pathogenic signaling pathways and therapeutic targets. Signal Transduct. Target Ther. 8, 56. 10.1038/s41392-023-01330-w 36737426 PMC9898571

[B80] Yin Z. Zhu W. Wu Q. Zhang Q. Guo S. Liu T. (2019). Glycyrrhizic acid suppresses osteoclast differentiation and postmenopausal osteoporosis by modulating the NF-κB, ERK, and JNK signaling pathways. Eur. J. Pharmacol. 859, 172550. 10.1016/j.ejphar.2019.172550 31323222

[B82] Zhang F. Shi X. Zhu Z. Feng L. Zhang Y. (2021). Experimental study on irisin targets TDP-43 to regulate p38MAPK pathway to relieve osteoarthritis. Anat. Res. 43 (06), 584–588. 10.3969/j.issn.1671-0770.2021.06.002

[B83] Zhang K. Barragan-Adjemian C. Ye L. Kotha S. Dallas M. Lu Y. (2006). E11/gp38 selective expression in osteocytes: regulation by mechanical strain and role in dendrite elongation. Mol. Cell Biol. 26 (12), 4539–4552. 10.1128/MCB.02120-05 16738320 PMC1489126

[B84] Zhang Y. He X. Xue Y. Jin Y. Wang K. Shi Q. (2024). Irisin alleviates palmitic acid-induced osteogenic inhibition in bone marrow mesenchymal stem cells. Chin. J. Tissue Eng. Res. 28(1):26–31. 10.12307/2023.772

[B85] Zhang Y. Li R. Meng Y. Li S. Donelan W. Zhao Y. (2014). Irisin stimulates browning of white adipocytes through mitogen-activated protein kinase p38 MAP kinase and ERK MAP kinase signaling. Diabetes 63 (2), 514–525. 10.2337/db13-1106 24150604 PMC13117908

[B86] Zhang Z. Li W. Xiong J. Qiu L. (2019). Effect of irisin on osteogenic differentiation of bone marrow mesenchymal stem cells under static force and mechanical strain. Jounral Chongqing Med. Univ. 44 (9), 1134–1139. 10.13406/j.cnki.cyxb.002006

[B87] Zhu J. Li J. Yao T. Li T. Chang B. Yi X. (2024). Analysis of the role of irisin receptor signaling in regulating osteogenic/adipogenic differentiation of bone marrow mesenchymal stem cells. Biotechnol. Genet. Eng. Rev. 40 (3), 2012–2035. 10.1080/02648725.2023.2197713 37010292

[B88] Zhu X. Li X. Wang X. Chen T. Tao F. Liu C. (2021). Irisin deficiency disturbs bone metabolism. J. Cell. physiology 236 (1), 664–676. 10.1002/jcp.29894 PMC7722136 32572964

